# A specific anti-citrullinated protein antibody profile identifies a group of rheumatoid arthritis patients with a toll-like receptor 4-mediated disease

**DOI:** 10.1186/s13075-016-1128-5

**Published:** 2016-10-06

**Authors:** Eric Hatterer, Limin Shang, Pierre Simonet, Suzanne Herren, Bruno Daubeuf, Stéphanie Teixeira, James Reilly, Greg Elson, Robert Nelson, Cem Gabay, Jeremy Sokolove, Iain B. McInnes, Marie Kosco-Vilbois, Walter Ferlin, Emmanuel Monnet, Cristina De Min

**Affiliations:** 1 NovImmune SA, 14 chemin des Aulx, 1228 Plan les Ouates, Switzerland; 2 University School of Medicine, Institute of infection, immunity and inflammation, 120 University Place, Glasgow, UK; 3 Geneva University Hospital, 26 avenue Beau-Sejour, 1211 Geneva, Switzerland; 4 Stanford University, 1000 Welch Rd Suite 203, Palo Alto, CA USA; 5 Present Address: Glenmark Pharmaceuticals SA, 5 chemin de la Combeta, 2300 La-Chaux-de-Fonds, Switzerland

**Keywords:** Rheumatoid arthritis, Toll-like receptor 4, Synovial gluid, Cytokines, ACPA, Monoclonal antibody

## Abstract

**Background:**

Increased expression of toll-like receptor 4 (TLR4) and its endogenous ligands, is characteristic of rheumatoid arthritis (RA) synovitis. In this study, we evaluated how these TLR4 ligands may drive pathogenic processes and whether the fine profiling of anti-citrullinated protein antibodies (ACPA) based on their target specificity might provide a simple means to predict therapeutic benefit when neutralizing TLR4 in this disease.

**Methods:**

The capacity of RA synovial fluids (RASF) to stimulate cytokine production in monocytes from patients with RA was analyzed by ELISA. The presence of TLR4 activators in RASF was determined by measuring the levels of ACPA, ACPA subtypes with reactivity to specific citrullinated peptides and other TLR4 ligands. Neutralization of TLR4 signaling was investigated using NI-0101, a therapeutic antibody that targets TLR4.

**Results:**

RASF exhibited a heterogeneous capacity to induce production of proinflammatory cytokines by monocytes isolated from patients with RA. Such cytokine responses were significantly modified by TLR4 blockade achieved using NI-0101. The analysis of the content of RASF and matched sera demonstrated that ACPA fine specificities in patient samples predict cellular response to anti-TLR4 exposure in vitro.

**Conclusion:**

TLR4 represents a possible therapeutic target in RA. Our study demonstrates that TLR4 inhibition in an ex vivo model of RA pathogenesis can significantly modulate cytokine release and does so in specific subgroups of RA patient-derived samples. It also suggests that ACPA fine profiling has the potential to identify RA patients with a predominantly TLR4-driven pathotype that could be used to predict preferential response to TLR4 antagonism.

**Electronic supplementary material:**

The online version of this article (doi:10.1186/s13075-016-1128-5) contains supplementary material, which is available to authorized users.

## Background

Synovial inflammation is a hallmark of RA. While proinflammatory cytokines are considered to have a pivotal role in the pathogenesis of RA [1], the factors responsible for the initiation and perpetuation of the complex cytokine response are still not completely understood. Toll-like receptors (TLRs) play an important role in the initiation of innate immune responses and in the instruction of the adaptive arms of immunity. Both components of immune effector function are evident in RA pathogenesis and coincide with the tissue lesion. In particular, by their capacity to recognize the endogenous ligands released during tissue damage TLRs are attractive candidates for the mediation of chronicity in RA [2–6].

TLR4 is a receptor for immune complexes containing citrullinated proteins, in particular citrullinated fibrinogen (cFb-IC). Anti-cFb antibodies are present in over 50 % of patients with RA [7, 8]. Furthermore, anti-citrullinated protein antibodies (ACPA) titers correspond with more aggressive RA progression [9]. The presence of ACPA is one of the classification criteria for RA. For these reasons, the potential role of a subgroup of ACPA/cit-peptide complexes with reactivity to TLR4 deserves to be investigated as a potential pathogenic pathway in RA. TLR4 deficiency or TLR4 pharmacological inhibition reduces disease progression in mouse models of RA [10, 11]. TLR4 is expressed both on macrophages and synovial fibroblasts in synovial membrane in RA, two key cell types implicated in cytokine production within the inflamed joint [12, 13]. Recently, it has been reported that myeloid cells from patients with RA demonstrate an exaggerated response to TLR4 agonism [14, 15].

The inflammation present in the joints, the primary site of inflammation in patient with RA, has been reported to be heterogeneous among individual patients at the cellular and molecular levels [16]. Thus, to further investigate the role of TLR4 and its endogenous ligands in RA pathogenesis, we evaluated levels of different TLR4 ligands in RA synovial fluid (RASF), including antibodies against citrullinated proteins and their potential association with TLR4-dependent inflammation. We examined the cytokine induction capacity of RASF with an in vitro bioassay that evaluated the production of cytokines by monocytes from patients with RA. The contribution of TLR4 activation to RASF-stimulated cytokine production was assessed in the presence of NI-0101, an anti-human TLR4 monoclonal antibody (mAb) [17]. We found correlation between TLR4-dependent cytokine induction and ACPA targeting specific citrullinated epitopes in synovial fluid (SF) and in serum from patients with RA.

## Methods

### Patients, RASF and paired sera samples

RASF samples from the inflamed joints of 40 patients with RA were obtained from the ethically approved Tissue Bank of the Universities of Glasgow and Geneva. RA was diagnosed according to the 1987 revised criteria of the American College of Rheumatology (ACR). At the time of fluid collection, the mean age of patients with RA was 62 years (range 46–77 years). Paired RA sera samples were collected from 22 of these patients: post-mortem SF samples from 4 subjects without RA (purchased from Asterand) were used as controls.

### RA monocyte preparation

Peripheral blood mononuclear cells (PBMCs) were isolated from heparinized whole blood obtained from patients with RA (n = 7) by Ficoll Paque (GE Healthcare) density gradient centrifugation. Isolated cells were suspended in complete medium containing RPMI medium supplemented with 10 % heat-inactivated fetal bovine serum, 2 mM L-glutamine, 50 U/mL penicillin, and 50 μg/mL streptomycin (Sigma). CD14^+^ monocytes were then separated from PBMCs using anti-CD14 mAb-coupled magnetic beads followed by MACS column separation according to the manufacturer’s protocol (Miltenyi Biotec). Flow cytometric analysis revealed that CD14^+^ monocyte cell fractions contained >85 % CD11b^+^ cells.

### Cell stimulation

Monocytes derived from patients with RA were plated in 96-well culture plates at 5.10^4^ cells/well. NI-0101, formerly designated as Hu 15C1 [17], or a human IgG_1_ isotype control (clone DA4) was added to wells at a final concentration of 20 μg/mL. Plates were incubated (30 minutes, 37 °C) prior to adding SF (pooled or individual) diluted to a final concentration of 2.5 %. Ultrapure lipopolysaccharide (LPS) (*Salmonella minnesota*), added at 10 ng/mL (List Biological Laboratories), was used as a positive control. After incubation (24 h, 37 °C), supernatants were harvested and cytokine levels measured by ELISA (eBiosciences) or multiplexing assays (Invitrogen).

### TLR4 ligand quantification and ACPA fine specificity assays

RASF were characterized for levels of ACPA (commercial anti-cyclic citrullinated peptide (CCP)-2 ELISA, Eurodiagnostica), HMGB1, and S100A8/A9 using commercial ELISA kits (Diagnostik Service GmbH, Hycult and IBL International, respectively) according to the manufacturers’ protocols. Antibody reactivity against the citrullinated form of linear peptides derived from fibrinogen and histone 2A were determined by ELISA. Sequences of citrullinated peptides used in the assay are presented in Additional file 1. Ninety six well plates (Nunc) were coated and incubated overnight with peptides in their native or citrullinated form, all at a concentration of 10 μg/mL. Plates were washed and blocked (1 h, 4 °C) with 2 % BSA in PBS. For the detection of specific IgG, RASF samples diluted 1:50 in 2 % BSA, 2 M NaCl in PBS were added and incubated (1 h, 4 °C). Plates were then washed prior to adding an anti-human IgG Fc-horseradish peroxidase (HRP) (Sigma; 1 h). Tetramethylbenzidine (Sigma) was added and the reaction stopped with 2 N H_2_SO_4_.

Plates were read at 450 nm using an ELISA plate reader (BioTek Instruments). The difference (delta) in optical density (OD) was calculated as the immunoreactivity against citrulline peptide minus the immunoreactivity against arginine control peptide. The data are shown as arbitrary units where the delta OD for each RASF sample was normalized by the threshold calculated with non-RA SF samples (set at 1, dashed line) above which a sample is considered positive.

### RA patient stratification

RASF samples from patients with RA were classified as NI-0101 responders (R) if NI-0101 was able to block (partially or totally) RASF-induced IL-6 production from RA monocytes. Others were classified as NI-0101 non-responders (NR). Correlation was tested between NI-0101 responder or non-responder samples from patients with RA and the presence of ACPA, specific ACPA fine profiles, and levels of TLR4 ligands (see Additional file 2). Endotoxin levels were lower than 1.22 EU/mL in the final concentration of the RASF samples measured using the Limulus amebocyte lysate (LAL) test. This level of endotoxin did not stimulate TLR4 activation of myeloid cells in an LPS dose response study (data not shown).

### Statistical analysis

The Mann-Whitney *U* test was used to compare difference between groups. Statistical significance is denoted as follows: ***p < 0.001, **p < 0.01, *p < 0.05.

## Results

### Synovial fluids from patients with RA contain activators that stimulate disease-relevant cell types through the TLR4 pathway

As RASF from some patients with RA contain endogenous TLR4 ligands, and as joint-infiltrating myeloid cells are a source of proinflammatory cytokines in RA, we first investigated whether the endogenous TLR4 ligands in RASF samples contribute to the inflammatory potential of RASF, by stimulating myeloid cells from RA donors. The limited volume of individual RASF samples used in this study was not sufficient to be tested with multiple RA monocyte donors for the stimulation of multiple cytokines. A pool of RASF samples was thus created using eight ACPA-positive RASF samples containing high levels of TLR4 ligands (see Additional file 3) to allow investigation of the broad inflammatory potential of TLR4 ligands in RASF samples with multiple RA myeloid cell donors. RASF samples with high levels of ACPA are expected to contain ACPA immune complexes, which have been reported as TLR4 ligands. The involvement of TLR4 signaling was assessed with a monoclonal antibody, NI-0101, which has been developed to bind human TLR4 and neutralize its activation. To test NI-0101 specificity for TLR4 versus other TLRs, a monocytic TLR reporter cell line was used in combination with the ligands for various TLRs.

**Fig. 1:**
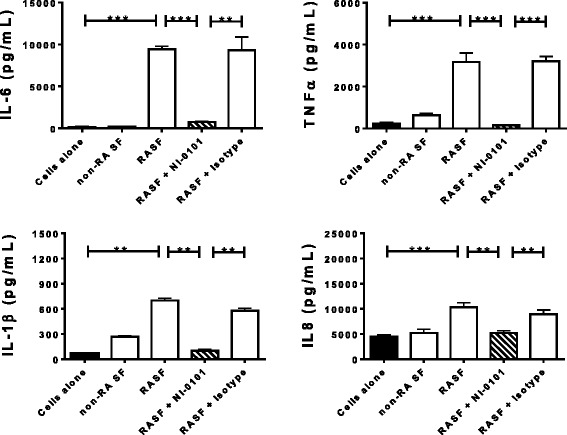
Interference in toll-like receptor 4 (TLR4) signaling blocks IL-6, TNFα, IL-1β and IL-8 production by monocytes stimulated by synovial fluid from patients with rheumatoid arthritis (*RASF*). TLR4 signaling was blocked with anti-human TLR4 monoclonal antibody, NI-0101. Representative data shown for monocytes obtained from one of seven patients with rheumatoid arthritis (*RA*). Data are presented as mean +/- SEM. The Mann-Whitney *U* test was performed to identify differences among groups: **p < 0.01, ***p < 0.001

NI-0101 blocked TLR4-induced activation but had no effect on other TLRs (see Additional file 4). Incubation with either LPS or the pooled RASF containing high levels of endogenous TLR4 ligands, in contrast to non-RA SF (pooled from four subjects without RA), stimulated a significant increase in IL-6 release from RA-derived monocytes from seven individual RA donors (see Additional file 5) (data not shown). Blocking TLR4 activation using NI-0101 impaired the increase in cytokine production, often bringing cytokine levels back to baseline (see Additional file 5). The capacity of RASF containing high levels of endogenous TLR4 ligands to induce IL-6 production and its blockade by NI-0101 was also observed using RA synovial fibroblasts (see Additional file 6). In addition, RASF-induced cytokine production was shown to be TNF-independent, as etanercept, a soluble TNF receptor (TNFR)-Fc fusion protein, had no impact on RASF-stimulated IL-6 production (see Additional file 6). The pooled RASF samples containing high levels of endogenous TLR4 ligands also induced significant production of TNFα, IL-1β and IL-8 (Fig. 1) by monocytes derived from patients with RA. Furthermore, the production of each cytokine was significantly reduced when TLR4 signaling was neutralized using NI-0101. These data suggest that RASF containing activators of the TLR4 pathway can play a role in stimulating immune and non-immune cells within inflamed joints.

**Fig. 2:**
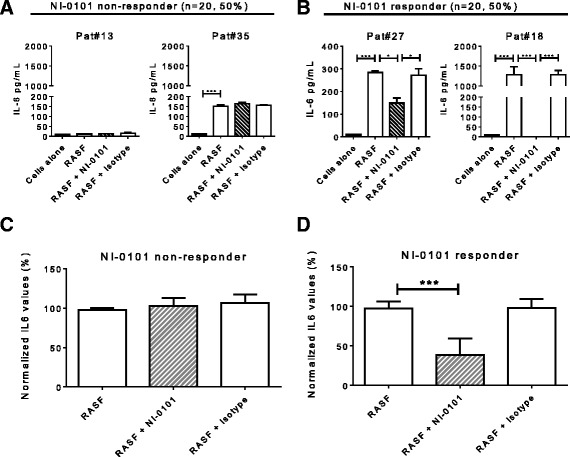
Heterogeneous capacity of rheumatoid arthritis (*RA*) synovial fluid (*RASF*) samples to stimulate cytokine production and respond to TLR4 blockade. RASF samples from patients (*Pat*) were classified as NI-0101 responders if NI-0101 was able to block (partially or totally) RASF-induced IL-6 production from RA monocytes. Others were classified as NI-0101 non-responders. **a**, **b** Representative examples of RASF from non-responders (*Pat#13*, *Pat*#35) and from responders (*Pat#27*, *Pat#18*). Of the 40 RASF samples tested, 20 were classified as NI-0101 responders (50 %) and 20 as NI-0101 non-responders (50 %). **c**, **d** Mean levels of IL-6 in RASF NI-0101 responder and non-responder RASF samples. IL-6 is presented as normalized values with IL-6 levels induced by RASF arbitrarily set as 100 % induction. RASF samples that were unable to induce IL-6 from RA monocytes but were impacted upon by NI-0101 are excluded from the non-responder group. The Mann-Whitney *U* test was performed to test for differences among groups; ***p < 0.001, *p < 0.05

As the pool of eight RASF samples containing high levels of endogenous TLR4 ligands provoked a potent inflammatory response, we next sought to investigate the ability of individual RASF samples containing different levels of endogenous TLR4 ligands to induce cytokine production and explore variability among patient donors. Using monocytes isolated from patients with RA, we observed that 29 out of 40 tested RASF samples stimulated IL-6 production, which was inhibited by the presence of NI-0101 in 20 of those 29 samples (Fig. 2a and b and Additional file 7). The RASF samples that did not stimulate IL-6 production and those that stimulated IL-6 production but did not respond to NI-0101 treatment were designated as NI-0101 non-responders (Fig. 2a). The RASF samples that stimulated IL-6 production and demonstrated responsiveness (partial or total) to NI-0101 treatment were designated as NI-0101 responders (Fig. 2b). In contrast to the lack of effect of NI-0101 on RASF-stimulated IL-6 production in the NI-0101 non-responder group (Fig. 2c), treatment with NI-0101 reduced RASF-induced IL-6 by a mean value of 61 % in NI-0101 responder samples (Fig. 2d). These results suggest that individual RASF can cause variable levels of TLR4-dependent cytokine production by monocytes from patients with RA.

### Expression of ACPA and TLR4 ligands in RASF samples and their correlation with TLR4 blockade of cytokine release

**Fig. 3:**
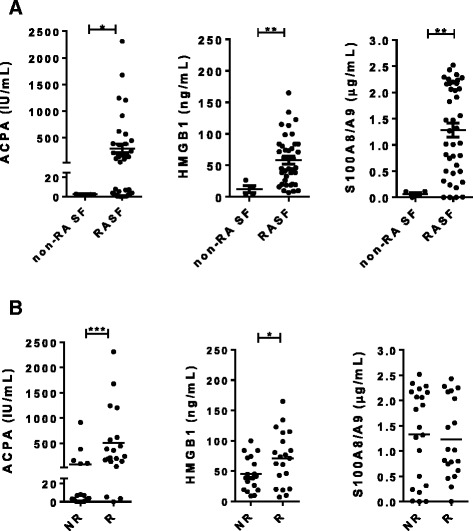
Expression of anti-citrullinated protein antibodies (*ACPA*) and ligands of toll-like receptor 4 in samples of synovial fluid (*SF*) from patients with rheumatoid arthritis (*RA*) and correlation between these and NI-0101 response. **a** Expression of ACPA, HMGB1 and S100A8/A9 in SF from subjects without RA (*non-RA SF*) (n = 4 samples) and patients with RA (*RASF*) (n = 40 samples). **b** Correlation between ACPA, HMGB1 or S100A8/A9 and NI-0101 response. RASF samples were classified as NI-0101 non-responders (*NR*) or NI-0101 responders (*R*) according to Fig. 2. The Mann-Whitney *U* test was performed to test for differences among groups; *p < 0.05, **p < 0.01

We next analyzed the presence of ACPA and several known TLR4 agonists including HMGB1 and S100A8/A9 in individual RASF samples. In contrast to non-RA SF samples, increased levels of ACPA and TLR4 ligands were detected in samples from patients with RA (Fig. 3a). Using 25 U/mL as the cutoff (the cutoff used in clinical practice), samples were divided into an ACPA-positive (n = 22) and an ACPA-negative (n = 18) subset. A continuous distribution was observed for the expression of S100A8/A9 and HMGB1 (Fig. 3a). To correlate the degree of cytokine inhibition by NI-0101 in individual RASF-induced cytokine production assays, with ACPA and TLR4 ligand levels in RASF, a sample was designated as non-responder or responder to NI-0101 blockade based on the ability of NI-0101 to decrease RASF-induced IL-6 production from RA monocytes (Fig. 2). Additional file 2 depicts the stratification of patients with RA from initial assessment of NI-0101 response to biomarker investigation. On average, ACPA and HMGB1 in the NI-0101 responder group were significantly higher than those in the NI-0101 non-responder group, whereas S100A8/A9 was similar between the two groups (Fig. 3b and Additional file 8).

### Autoantibodies against specific citrullinated peptides are associated with NI-0101 response

**Fig. 4:**
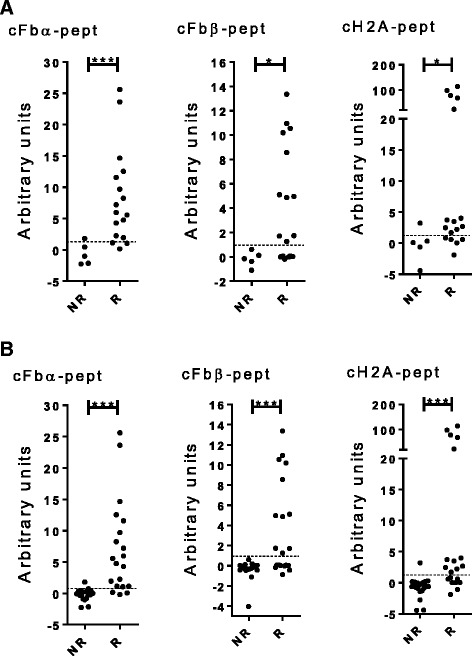
Anti-citrullinated protein antibodies (ACPA) fine specificity in the synovial fluid samples of patients with rheumatoid arthritis and their correlation with NI-0101 response. Antibody reactivity against the citrullinated peptides derived from fibrinogen-α (cFbα − pept), fibrinogen-β (*cFbβ-pept*) and histone-2A (*cH2A-pept*) were determined by ELISA and expression in synovial fluids was measured in ACPA-positive rheumatoid arthritis synovial fluid (RASF) samples (**a**) (n = 22) and both ACPA-negative and ACPA-positive RASF samples (**b**) (n = 40). RASF samples were classified as NI-0101 non-responders (*NR*) or NI-0101 responders (*R*). The Mann-Whitney *U* test was performed to compare observed changes; ***p < 0.001, **p < 0.01, *p < 0.05

Interestingly, there was association with inhibition by NI-0101 in 17 out of 22 ACPA-positive RASF samples, but not in the remaining 5 samples. As ACPA from an individual patient comprise a mixture of antibodies recognizing different citrullinated proteins, e.g., fibrinogen, histone 2A and fibronectin [18], we hypothesized that the citrullinated proteins characterizing ACPA in a given patient in the NI-0101-sensitive group may be different from those in the non-responder group. Therefore, ACPA-positive RASF samples were further classified according to reactivity to individual citrullinated peptides from different proteins. Samples positive for reactivity to citrullinated peptides derived from the α-chains and β-chains of fibrinogen (cFbα-pept and cFbβ-pept) and histone 2A (cH2A-pept) were represented significantly more often in the NI-0101 responder group than in the non-responder group (Fig. 4a). To determine the relative predictive power of different citrullinated peptides to identify NI-0101 responders, we assessed the sensitivity and specificity of individual peptides and different combinations of the peptides (Table 1). cFbα peptide was the strongest predictor among the individual peptides, with sensitivity of 94 % and specificity of 80 %. Combining anti-cFbα-derived and anti-cH2A-derived peptides increased the sensitivity to 100 % with specificity of 80 % (Table 1). Adding to the combination the reactivity to cFbβ did not further improve the predictive power.

**Table 1:** The sensitivity and specificity of individual citrullinated peptides and their combinations in predicting NI-0101 response

	ACPA-positive RASF		ACPA-negative and ACPA-positive RASF	
ACPA-related biomarkers	Sensitivity	Specificity	Sensitivity	Specificity
ACPA (CCP2)	N/A	N/A	85 %	75 %
cFbα-peptide (#1)	94 %	80 %	85 %	95 %
cFbβ-peptide (#2)	65 %	100 %	55 %	100 %
cH2A-peptide (#3)	70 %	80 %	60 %	95 %
(#1) + (#2)	94 %	80 %	85 %	95 %
(#1) + (#3)	100 %	80 %	90 %	95 %
(#2) + (#3)	88 %	80 %	75 %	95 %
(#1) + (#2) + (#3)	100 %	80 %	90 %	95 %

Sensitivity in this context is defined as the percentage of NI-0101 responders identified as positive in the assay and specificity as the percentage of NI-0101 non-responders identified as negative in the assay. Sensitivity and specificity were determined in the following groups: (i) anti-citrullinated protein antibodies (ACPA)-positive rheumatoid arthritis synovial fluid (RASF) samples (n = 22), (ii) ACPA-negative and ACPA-positive RASF samples (n = 40). *N/A* not applicable, *CCP2* anti-cyclic citrullinated peptide-2

We next assessed the capacity of the citrullinated peptides to predict responders to NI-0101 independently of ACPA positivity. Similar to the ACPA-positive samples, when both ACPA-positive and ACPA-negative RASF samples were included, all samples positive for reactivity to cFbβ peptide and the majority of samples positive for reactivity to cFbα or cH2A peptides were in the NI-0101 responder group (Fig. 4b). cFbα peptide was the strongest predictor among the individual peptides, with sensitivity of 85 % and specificity of 95 %. In comparison, ACPA positivity predicted NI-0101 responders with sensitivity of 85 % and specificity of 75 %. Similar to the APCA-positive samples, combining the results of the anti-cFbα-derived and anti-cH2A-derived peptides provided the highest sensitivity and specificity to predict NI-0101 response with sensitivity of 90 % and specificity of 95 % (Table 1).

In order to further characterize these potential predictive biomarkers of patients’ responses to NI-0101, we performed receiver operating characteristic (ROC) analysis. Additional file 9 shows detailed sensitivity and specificity data with the 95 % confidence interval, for the three citrullinated peptides. The area under the ROC curve was 0.83 for CCP2 and 0.93, 0.86 and 0.95 for cFbα-pept, cH2A-pept and the combination of both peptides, respectively. These results demonstrated that reactivity to a specific set of citrullinated peptides predicted the response to NI-0101 better than ACPA positivity alone (based on the anti-CCP2 test).

### ACPA fine profiling in serum samples from patients with RA and its correlation with the capacity for in vitro inhibition by NI-0101 in their paired RASF

**Fig. 5:**
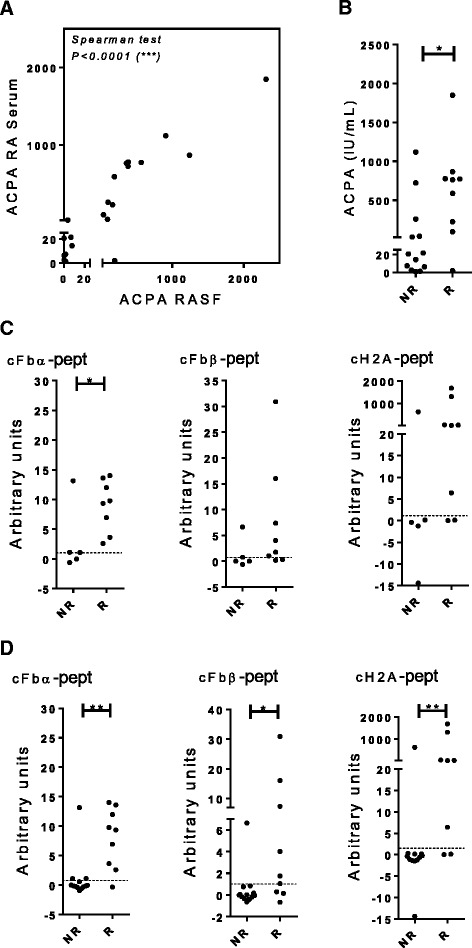
Anti-citrullinated protein antibodies (*ACPA*) fine specificity in paired sera samples of patients with rheumatoid arthritis (RA) and their correlation with RA synovial fluid (*RASF*) response to NI-0101. **a** Correlation between ACPA in paired samples of sera from patients with RA and synovial fluids (n = 22). **b** ACPA in paired samples of sera from patients with RA classified according to RASF response to NI-0101 (NI-0101 non-responders (*NR*) or NI-0101 responders (*R*)). **c**, **d** Antibody reactivity against the citrullinated peptides derived from fibrinogen-α (*cFbα-pept*), fibrinogen-β (*cFbβ-pept*) and histone-2A (*cH2A-pept*) were determined by ELISA in paired samples of sera from patients with RA and correlation with response to NI-0101 was tested. **c** Paired sera samples from ACPA-positive patients with RA (n = 13). **d** Paired samples of sera from from ACPA-positive and ACPA-negative patients with RA (n = 22). The Mann-Whitney *U* test was performed to compare observed changes; **p < 0.01, *p < 0.05

Paired RA serum and RASF samples were obtained from 22 patients with RA. ACPA positivity in RASF correlated with positivity in the paired serum samples (Fig. 5a), suggesting that ACPA profiles in serum reflect those in the joint. ACPA positivity in the sera was associated with a clear inhibition of cytokine production by NI-0101 in matched RASF samples. Interestingly 8 out of the 13 ACPA-positive paired RA serum samples were associated with NI-0101 response, whereas 5 were not (Fig. 5b). Therefore, ACPA-positive paired RA serum samples were further classified according to reactivity to individual citrullinated peptides from different proteins. Samples positive for reactivity to cFbα-pept were significantly more frequent in the NI-0101 responder group than in the non-responder group (Fig. 5c). A trend for presence of cFbβ-derived and cH2A-derived peptides was observed (Fig. 5c). We next assessed the capacity of specific citrullinated peptides to predict NI-0101 responders independently of ACPA positivity. The majority of samples positive for reactivity to cFbα, cFbβ or cH2A peptides were significantly associated with the NI-0101 responder group (Fig. 5d).

## Discussion

Genetics, environmental risk factors, autoantibody patterns and cytokine profiles described in RA are commensurate with the well-recognized clinical heterogeneity of this disease. Unfortunately, there is no biomarker available to predict the response of an individual patient with RA to current therapies. This has an impact on quality of care and increases the financial burden. It may also expose patients to drug-related risk with no tangible benefit. Thus, the identification of therapeutic agents targeting new pathogenic pathways and identifying biomarkers to predict patient response to these agents may respond to the unmet needs in RA.

In the present study, we demonstrated that RASF-stimulated cytokine production in a subgroup of patients with RA was mediated through TLR4 activation. In particular, ACPA antibodies reactive to a set of specific citrullinated peptides and several TLR4 ligands were expressed heterogeneously in the RASF samples and their positivity and levels were correlated with the ability of the RASF samples to stimulate inflammatory cytokine production. We further demonstrated that ACPA fine profiles in sera predict RASF-stimulated TLR4 activation. These data demonstrate the possible pathogenic role of TLR4 activation in a subgroup of patients with RA and that the relative levels of its ligands may represent potential biomarkers to predict patient responses to NI-0101.

RA is a heterogeneous autoimmune disease with the different pathways involved leading to joint inflammation. Synovial fluid represents the complex microenvironment in the synovium. In particular, several TLR4 ligands may contribute to RASF-induced cytokine production, as many TLR4 activators have been described in synovial tissues [1, 5, 19–21]. Several TLRs, including TLR2 and TLR4, have been suggested to play a role in joint inflammation. The idea of the involvement of TLR2 in RA is supported by enhanced expression of TLR2 in synovial lining cells [22–26] and elevated levels of TLR2 ligands in synovial tissues in RA [27﻿, 28﻿, 29–31]. In addition, an anti-TLR2 antibody has recently been demonstrated to decrease inflammation in a mouse model of arthritis [32], and blockade of TLR2 to reduce spontaneous cytokine release from synovial explant cultures in RA [33].

Interestingly, our data demonstrated that RASF-stimulated cytokine production in RA monocytes was almost totally reversed by NI-0101 exposure in some HMGB1-positive samples. These results suggest that TLR4 may be required for the endogenous ligands in RASF to induce proinflammatory cytokine production and that TLR4 blockade alone can prevent the cytokine production. Consistent with this observation, we demonstrated that interfering with TLR4 signaling alone is sufficient to block synergistic IL-6 induction from monocytes stimulated by the combined TLR2 and TLR4 activation (see Additional file 10). Our data point to a pathogenic role for multiple TLR4 ligands in joint inflammation in a subgroup of patients with RA, and demonstrate that NI-0101 can effectively block these pathogenic ligands. Because of the multiplicity of TLR4 ligands in RA, direct blockade of the receptor activation independent of ligands likely affords a more promising strategy than inhibiting a single ligand of TLR4. In this aspect, NI-0101 has been demonstrated to be a ligand-independent TLR4 blocker and thus, well-positioned to block TLR4 activation in the subgroup of patients with increased expression of TLR4 ligands [17].

Among the different types of autoantibodies, ACPA are highly specific to RA and are reported as being pathogenic and associated with the most severe and erosive forms of the disease [34–37]. The mechanism by which ACPA promote inflammation is proposed to be related to ACPA activating complement, abnormal glycosylation pattern or IC formed by ACPA and citrullinated proteins, resulting in a higher affinity for Fc gamma receptors (FcγRs) [7, 38–42]. Interestingly, in the present study, we observed that TLR4-dependent cytokine induction was more frequent in ACPA-positive compared to ACPA-negative samples, suggesting that the level of ACPA may dictate the response of individual patients with RA to TLR4 blockade.

Our data also demonstrated that ACPA reactivity to citrullinated Fbα-derived, Fbβ-derived and H2A-derived peptides is associated with NI-0101 response. Furthermore, combining positivity for anti-cFbα and -cH2A antibodies provides the best classifier to predict NI-0101 response (data not shown). Although ACPA alone has not been demonstrated to be TLR4 ligands, ACPA forms immune complexes containing antigens with innate immunostimulatory capacity (i.e., cFb and citrullinated histones), which have recently been reported to be a TLR4 ligand [7, 43–45]. The presence of cFb-IC, cHistones-IC and other TLR4-activating immune complexes containing ACPA in the RASF samples may explain the association between ACPA positivity in those samples and their response to TLR4 blockade. In support of the notion that cFb-IC have a role in TLR4-driven joint inflammation, we observed that the profiling of ACPA-positive RASF and paired serum samples from patients with RA, by their reactivity to cFbα and cFbβ chains, identified the TLR4-dependent subgroup with a greater specificity than ACPA alone. Similarly, histones are reported to enhance LPS-induced cytokine production, further strengthening the central role of TLR4. Also, more recently, citrullination of H2A and H2B were demonstrated to enhance their capacity to stimulate proinflammatory cytokine production [43, 46]. Therefore, the presence of anti-cH2A antibodies in NI-0101 responder RASF may reflect the presence of a synergistic effect of cH2A or ACPA complexed to cH2A with other TLR4 ligands on TLR4-driven cytokine production. Furthermore, when NI-0101 non-responder and partial responder RASF samples (defined by inhibition of IL-6 induction <50 %) are grouped together and compared to the NI-0101 responder group (defined by inhibition of IL-6 induction >50 %), the levels of two of the three anti-cit peptide antibodies (i.e., cFbα-pept and cFbβ-pept) are significantly lower in the non-responder and partial-responder group (data not shown). These results suggest that a partial response to NI-0101 may be associated with the presence of ligands to other TLRs. Our data shed new light on the role of ACPA reactivity, which may allow us to identify ACPA-positive RASF with respect to ability to mediate TLR4-driven cytokine production.

cFb-IC and cH2A-IC stimulate macrophage activation through a mechanism involving both TLR4 and FcγRs [7, 43]. The co-engagement of TLR4 and FcγRs may increase the avidity of cFb-IC binding to the cell surface and enhance its capacity to activate TLR4. Interestingly, the NI-0101 mechanism of action involves the co-engagement of TLR4 and FcγRs to increase its own binding avidity and inhibitory capacity [17]. This mechanism of action is therefore well-fitted to block cFb-IC and other immune-complex-induced TLR4 activation, as it not only inhibits TLR4 signaling by blocking receptor dimerization, but also has the potential to reduce the binding avidity of cFb-IC to the cell surface by competing for FcγR binding. Indeed, we demonstrated that the Fc-dependent mechanism of action rendered NI-0101 a more effective antibody in inhibiting cFb-IC-stimulated TNF-α production from macrophages (see Additional file 11). Similar data were generated using H2B-IC stimulated macrophages (data not shown). The mechanism of action of cFb-IC also suggests that it is more effective in activating TLR4 on FcγR-bearing cells than on FcγR-negative cells.

Taken together, this study demonstrates in vitro the contribution of TLR4 to the inflammatory process in specific subgroups of patients with RA, and that TLR4-induced cytokine production can be blocked by a novel therapeutic anti-human TLR4 antibody, NI-0101. The results highlight TLR4 as a potential new therapeutic target in RA and portend the use of TLR4 ligands and ACPA fine profiles as biomarkers to predict the response of individual patients with RA to TLR4 inhibitors. If confirmed in clinical settings, ACPA specificities would define a subpopulation of patients with RA, characterized with TLR4-mediated disease.

## Conclusions

In summary, the data presented herein demonstrate TLR4-dependent proinflammatory cytokine production in samples from patients with RA, and its correlation with the levels of TLR4 ligands in RASF and paired serum samples. In particular, we showed that immune complexes containing a subset of ACPA bound to their specific autoantigens can activate cells via TLR4 and the presence of these specific ACPA in patient RASF and paired samples of sera predicts cellular response to anti-TLR4 exposure in vitro. Furthermore, this study suggests that ACPA fine profiling in blood could be used as biomarkers to identify patients with RA who could benefit from TLR4-targeted therapy. These findings have been the foundation to design the proof-of-concept clinical trial in ACPA-positive patients with RA, aimed at validating the therapeutic potential of a new first-in-class anti-TLR4 monoclonal antibody, NI-0101, and the potential of predictive biomarkers (i.e., TLR4 ligands and in particular, levels of ACPA subtypes in serum) to identify patients more likely to benefit from TLR4 blockade by NI-0101. The clinical study has recently been approved by the Food and Drug Agency (FDA) and Medicines and Healthcare Products Regulatory Agency (MHRA).

## Additional files

Additional file 1: Citrullinated peptide sequences used to characterize ACPA specificities in RA synovial fluid and paired serum samples. Sequences of citrullinated peptides used in the assay. (DOCX 16 kb)
Additional file 2: Methodological approach used to identify NI-0101 responder samples and biomarkers. RA patient stratification methodology. (DOCX 27 kb)
Additional file 3: The levels of ACPA, HMGB1 and S100A8/A9 in individual RASF samples pooled to create a single sample then used to stimulate RA monocytes. Characterization of the pool of RASF samples created with eight ACPA-positive RASF samples containing high levels of TLR4 ligands. (DOCX 16 kb)
Additional file 4: NI-0101 specifically blocked human TLR4 but not other members of the TLR family. NI-0101 blocked TLR4-induced activation while having no effect on other TLRs. (DOCX 804 kb)
Additional file 5: The inhibition by NI-0101 of IL-6 production in monocytes obtained from seven patients with RA. Incubation with the pooled RASF containing high levels of endogenous TLR4 ligands stimulated a significant increase in IL-6 release from RA-derived monocytes from seven individual donors with RA. (DOCX 439 kb)
Additional file 6: Interfering with TLR4 signaling blocks IL-6 production RASF stimulated RA monocytes and RA synovial fibroblasts. Interfering with TLR4 signaling blocks IL-6 production from LPS or RASF-stimulated RA monocytes and RA synovial fibroblasts. (DOCX 272 kb)
Additional file 7: Stimulation of IL-6 production by individual RASF samples and their response to NI-0101 treatment. These results suggest that individual RASF can cause variable levels of TLR4-dependent cytokine production by monocytes from patients with RA. (DOCX 1399 kb)
Additional file 8: The levels of ACPA, HMGB1, S100A8/A9 in individual RASF samples from 40 patients with RA (*Pat*) and the capacity of these samples to respond to NI-0101 treatment in RA monocytes. RASF samples were classified as NI-0101 responders (*R*) or NI-0101 non-responders (*NR*). The average levels of ACPA and HMGB1 in the NI-0101 responder group were significantly higher than those in the NI-0101 non-responder group. (DOCX 369 kb)
Additional file 9: Receiver operating characteristic (ROC) curve analyses of anti-citrullinated peptides levels in RASF in predicting NI-0101 response. Detailed sensitivity and specificity levels with 95 % confidence interval of the 3 citrullinated peptides. (DOCX 20 kb)
Additional file 10: Stimulation with TLR2 and TLR4 ligands. Interfering with TLR4 signaling alone is sufficient to block synergistic IL-6 induction from monocytes stimulated by the combined TLR2 and TLR4 activation. (DOCX 84 kb)
Additional file 11: cFb-IC-induced TNF-α production was inhibited by TLR4 blockade with NI-0101. Fc-dependent mechanism of action rendered NI-0101 a more effective antibody in inhibiting cFb-IC-stimulated TNF-α production from macrophages. (DOCX 150 kb)

## Abbreviations

ACPA: Anti-citrullinated protein antibodies
ACR: American College of Rheumatology
AUC: Area under the curve
BSA: Bovine serum albumin
CCP-2: Anti-cyclic citrullinated peptide-2
cFb-IC: Immune complexes containing citrullinated fibrinogen
cFbα: α-chain of citrullinated fibrinogen
cFbβ: β-chain of citrullinated fibrinogen
cH2A: Citrullinated histone 2A
cH2B-IC: Immune complexes containing citrullinated histone 2B
ELISA: Enzyme-linked immunosorbent assay
FcγRs: Fc gamma receptors
HMGB1: High mobility group box 1
HRP: Horseradish peroxidise
IgG: Immunoglobulin G
IL: Interleukin
LPS: Lipopolysaccharide
mAb: Monoclonal antibody
OD: Optical density
PBMC: Peripheral blood mononuclear cells
PBS: Phosphate-buffered saline
pept: Peptide
RA: Rheumatoid arthritis
RASF: RA synovial fluid
ROC: Receiver operating characteristic
RPMI: Roswell Park Memorial Hospital
TLR: Toll-like receptor
TNF: Tumor necrosis factor
