# Supplementary material for: A specific anti-citrullinated protein antibody profile identifies a group of rheumatoid arthritis patients with a toll-like receptor 4-mediated disease
Source: Arthritis Res Ther. 2016 Oct 6;18:224. doi: 10.1186/s13075-016-1128-5 (PMC5053084; doi:10.1186/s13075-016-1128-5)
Supplement: Additional file 1: — Citrullinated peptide sequences used to characterize ACPA specificities in RA synovial fluid and paired serum samples. Sequences of citrullinated peptides used in the assay. (DOCX 16 kb) [file 13075_2016_1128_MOESM1_ESM.docx]

**Additional file 1**: Citrullinated peptide sequences used to characterize ACPA specificities in RA synovial fluid and paired serum samples

| Protein | Peptides | | Sequences |
| --- | --- | --- | --- |
| Fibrinogen | | cFbα-pept | NTKESSSHHPGIAEFPS-Cit-GK |
|  |  | cFbβ-pept | HHPGIAEFPS-Cit-GKSSSYSKQF |
| Histone-2A | | cH2A-pept | MSG-Cit-GKQGGKA-Cit-AKAKS-Cit-SS |
