# Supplementary material for: A specific anti-citrullinated protein antibody profile identifies a group of rheumatoid arthritis patients with a toll-like receptor 4-mediated disease
Source: Arthritis Res Ther. 2016 Oct 6;18:224. doi: 10.1186/s13075-016-1128-5 (PMC5053084; doi:10.1186/s13075-016-1128-5)
Supplement: Additional file 2: — Methodological approach used to identify NI-0101 responder samples and biomarkers. RA patient stratification methodology. (DOCX 27 kb) [file 13075_2016_1128_MOESM2_ESM.docx]

**Additional file 2**

**A- Classification of RA monocytes response to RASF and NI-0101**

RASF samples (n=40)

Assess cytokine induction by RASF and its blockade by NI-0101

NI-0101 responder (n=20)

NI-0101 non responder (n=20)

ACPA levels

ACPA +

TLR4 ligand levels

ACPA fine profile

Specificities tested against citrullinated peptides

Cytokine induction by RASF with partial or total inhibition

by NI-0101

No cytokine induction by RASF or cytokine induction with no inhibition by NI-0101

**B- Biomarkers correlation with NI-0101 response**

ACPA -

Correlation with NI-0101 response

RASF samples (n=40)

**Additional file 2:** Methodological approach used to identify NI-0101 responder samples and biomarkers
