# Supplementary material for: A specific anti-citrullinated protein antibody profile identifies a group of rheumatoid arthritis patients with a toll-like receptor 4-mediated disease
Source: Arthritis Res Ther. 2016 Oct 6;18:224. doi: 10.1186/s13075-016-1128-5 (PMC5053084; doi:10.1186/s13075-016-1128-5)
Supplement: Additional file 3: — The levels of ACPA, HMGB1 and S100A8/A9 in individual RASF samples pooled to create a single sample then used to stimulate RA monocytes. Characterization of the pool of RASF samples created with eight ACPA-positive RASF samples containing high levels of TLR4 ligands. (DOCX 16 kb) [file 13075_2016_1128_MOESM3_ESM.docx]

**Additional file 3:** The levels of ACPA, HMGB1 and S100A8/A9 in individual RASF samples pooled to create a single sample then used to stimulate RA monocytes.

| **RA Patient sample ID** | **ACPA (IU/mL)** | **HMGB1 (ng/mL)** | **S100A8/A9 (ng/mL)** |
| --- | --- | --- | --- |
| Pat#1 | 443.74 | 165.15 | 784 |
| RASF#2551 | 226.92 | 68.58 | 1018.5 |
| Pat#4 | 616.38 | 134.24 | 743.1 |
| RASF#2847 | 20.02 | 125.08 | 609.2 |
| Pat#8 | 142.69 | 122.85 | 798.1 |
| RASF#3731 | 182.44 | 83.37 | 804.4 |
| Pat#10 | 244.06 | 113.51 | 459.3 |
| Pat#11 | 213 | 98.64 | 813.3 |
| **Mean values of the pool** | **261.15** | **113.92** | **753.73** |
