# Supplementary material for: A specific anti-citrullinated protein antibody profile identifies a group of rheumatoid arthritis patients with a toll-like receptor 4-mediated disease
Source: Arthritis Res Ther. 2016 Oct 6;18:224. doi: 10.1186/s13075-016-1128-5 (PMC5053084; doi:10.1186/s13075-016-1128-5)
Supplement: Additional file 4: — NI-0101 specifically blocked human TLR4 but not other members of the TLR family. NI-0101 blocked TLR4-induced activation while having no effect on other TLRs. (DOCX 804 kb) [file 13075_2016_1128_MOESM4_ESM.docx]

**Additional file 4**

**Additional file 4:** NI-0101 specifically blocked human TLR4 but not other members of the TLR family. A: THP-1 CD14/Blue reporter cells were incubated with the indicated concentration of TLR ligands, which were pre-incubated with or without polymyxin B (100 μg/mL, 24 hours). B. Reporter cells were incubated with indicated concentration of NI-0101 in the presence of different TLR ligands Absorbance at 650 nm was then measured as an indicator for NF-kB activation.
