# Supplementary material for: A specific anti-citrullinated protein antibody profile identifies a group of rheumatoid arthritis patients with a toll-like receptor 4-mediated disease
Source: Arthritis Res Ther. 2016 Oct 6;18:224. doi: 10.1186/s13075-016-1128-5 (PMC5053084; doi:10.1186/s13075-016-1128-5)
Supplement: Additional file 5: — The inhibition by NI-0101 of IL-6 production in monocytes obtained from seven patients with RA. Incubation with the pooled RASF containing high levels of endogenous TLR4 ligands stimulated a significant increase in IL-6 release from RA-derived monocytes from seven individual donors with RA. (DOCX 439 kb) [file 13075_2016_1128_MOESM5_ESM.docx]

**Additional file 5**

**Additional file 5:** The inhibition by NI-0101 of IL-6 production in monocytes obtained from seven RA patients (A-G) stimulated by pooled RASF samples. Each condition was tested in triplicate. Data are presented as mean +/- SEM. Student’s t test performed to compare changes observed. *** p<0.001, ** p<0.01, * p<0.05.
