# Supplementary material for: A specific anti-citrullinated protein antibody profile identifies a group of rheumatoid arthritis patients with a toll-like receptor 4-mediated disease
Source: Arthritis Res Ther. 2016 Oct 6;18:224. doi: 10.1186/s13075-016-1128-5 (PMC5053084; doi:10.1186/s13075-016-1128-5)
Supplement: Additional file 6: — Interfering with TLR4 signaling blocks IL-6 production RASF stimulated RA monocytes and RA synovial fibroblasts. Interfering with TLR4 signaling blocks IL-6 production from LPS or RASF-stimulated RA monocytes and RA synovial fibroblasts. (DOCX 272 kb) [file 13075_2016_1128_MOESM6_ESM.docx]

**Additional file 6**

**Additional file 6:** Interfering with TLR4 signaling blocks IL-6 production from LPS- (A, B) or RASF (C, D) stimulated RA monocytes (A) and RA synovial fibroblasts (B, C, D). Representative data shown for monocytes obtained from 1 of 7 RA patient donors and for synovial fibroblasts obtained from 1 of 3 RA patient donors. Cells were either untreated (Cells alone) or preincubated with 20 ug/ml NI-0101 or isotype control for 30 min before the addition of 10 ng/ml LPS (A, B), pooled (n=4) non-RA synovial fluid samples (non-RASF; C) or pooled (n=8) RA synovial fluid samples (RASF; C, D). In some condition, RASF were mixed 1:1 during 30 min at 37°C with Enbrel (5 μg/mL) to inhibit endogenous TNFα (D). Each condition was tested in triplicate. Supernatants were collected 24 h post stimulation and IL-6 analyzed by ELISA. Data are presented as mean +/- SEM. The Mann Whitney’s U test was used to compare different groups. *** P < 0.001, ** P < 0.01.
