# Supplementary material for: A specific anti-citrullinated protein antibody profile identifies a group of rheumatoid arthritis patients with a toll-like receptor 4-mediated disease
Source: Arthritis Res Ther. 2016 Oct 6;18:224. doi: 10.1186/s13075-016-1128-5 (PMC5053084; doi:10.1186/s13075-016-1128-5)
Supplement: Additional file 7: — Stimulation of IL-6 production by individual RASF samples and their response to NI-0101 treatment. These results suggest that individual RASF can cause variable levels of TLR4-dependent cytokine production by monocytes from patients with RA. (DOCX 1399 kb) [file 13075_2016_1128_MOESM7_ESM.docx]

**Additional file 7**

**Additional file 7: Stimulation of IL-6 production by individual RASF samples and their response to NI-0101 treatment.** Heterogeneity exists among synovial fluid samples from individual RA patients. Representative data shown for monocytes obtained from 1 of 2 RA donors. Cells were either untreated (Cells alone) or preincubated with 20 ug/ml NI-0101 or isotype control for 30 min then stimulated by individual synovial fluid samples obtained from 36 RA patients (Pat). Each condition was tested in triplicate. Data are represented mean +/- SEM. Mann Whitney’s U test was performed to compare changes observed. *** p<0.001, ** p<0.01, * p<0.05.
