# Supplementary material for: A specific anti-citrullinated protein antibody profile identifies a group of rheumatoid arthritis patients with a toll-like receptor 4-mediated disease
Source: Arthritis Res Ther. 2016 Oct 6;18:224. doi: 10.1186/s13075-016-1128-5 (PMC5053084; doi:10.1186/s13075-016-1128-5)
Supplement: Additional file 8: — The levels of ACPA, HMGB1, S100A8/A9 in individual RASF samples from 40 patients with RA (Pat) and the capacity of these samples to respond to NI-0101 treatment in RA monocytes. RASF samples were classified as NI-0101 responders (R) or NI-0101 non-responders (NR). The average levels of ACPA and HMGB1 in the NI-0101 responder group were significantly higher than those in the NI-0101 non-responder group. (DOCX 369 kb) [file 13075_2016_1128_MOESM8_ESM.docx]

**Additional file 9**

|  | AUC | (95% IC) | |  |
| --- | --- | --- | --- | --- |
| ACPA (CCP2) | 0.83 | 0.70 to 0.97 |  |  |
| cFbα-peptide (#1) | 0.93 | 0.85 to 1.00 | | |
| cFbβ-peptide (#2) | 0.83 | 0.69 to 0.96 | | |
| cH2A-peptide (#3) | 0.86 | 0.74 to 0.98 | | |
| (#1) + (#3) | 0.95 | 0.87 to 1.02 | | |

**Additional file 9:** Receiver Operating Characteristic (ROC) curves analyses of anti-citrullinated peptides levels in RASF in predicting NI-0101 response. Receiver-operating characteristic (ROC) analysis of the detection of ACPA (A), anti-cFibα-pept (B), cFibβ-pept (C), cH2A-pept (D) and combination of cFibα-pept and cH2A-pept (E) obtained with 40 RASF samples. The predictive value of each citrullinated peptide at indentifying NI-0101 response is determined by calculating the area under the curve (AUC, F). The areas under the curve (AUC) values were calculated employing ROC analysis using the GraphPad software.
