# Supplementary material for: A specific anti-citrullinated protein antibody profile identifies a group of rheumatoid arthritis patients with a toll-like receptor 4-mediated disease
Source: Arthritis Res Ther. 2016 Oct 6;18:224. doi: 10.1186/s13075-016-1128-5 (PMC5053084; doi:10.1186/s13075-016-1128-5)
Supplement: Additional file 9: — Receiver operating characteristic (ROC) curve analyses of anti-citrullinated peptides levels in RASF in predicting NI-0101 response. Detailed sensitivity and specificity levels with 95 % confidence interval of the 3 citrullinated peptides. (DOCX 20 kb) [file 13075_2016_1128_MOESM9_ESM.docx]

**Additional file 8:** The levels of ACPA, HMGB1, S100A8/A9 in individual RASF samples from 40 RA patients (Pat) and the capacity of these samples to respond to NI-0101 treatment in RA monocytes. RASF samples were classified as NI-0101 responders (R) or NI-0101 non-responders (NR).

| Patient ID | NI-0101 response | ACPA  (IU/mL) | HMGB1  (ng/mL) | S100A8/A9  (μg/mL) |
| --- | --- | --- | --- | --- |
| Pat#1 | R | 443.7 | 165.15 | 0.78 |
| Pat#2 | R | 3.5 | 115.17 | 0.28 |
| Pat#3 | R | 138.9 | 71.07 | 1.03 |
| Pat#4 | R | 616.4 | 134.24 | 0.74 |
| Pat#5 | NR | 2.6 | 99.81 | 0.18 |
| Pat#6 | NR | 7.1 | 18.73 | 0.01 |
| Pat#7 | R | 5.1 | 46.23 | 0.61 |
| Pat#8 | R | 142.7 | 122.85 | 0.79 |
| Pat#9 | NR | 2.4 | 9.79 | 0.00 |
| Pat#10 | R | 244.1 | 113.51 | 0.45 |
| Pat#11 | R | 213.0 | 98.64 | 0.81 |
| Pat#12 | NR | 3.0 | 44.76 | 0.31 |
| Pat#13 | NR | 159.7 | nd | 2.34 |
| Pat#14 | R | 197.6 | 20.32 | 2.17 |
| Pat#15 | NR | 910.5 | 38.31 | 1.01 |
| Pat#16 | R | 1242.6 | 80.5 | 2.25 |
| Pat#17 | NR | 104.8 | 15.96 | 2.52 |
| Pat#18 | R | 41.1 | 19.92 | 1.02 |
| Pat#19 | NR | 1.0 | 19.28 | 2.16 |
| Pat#20 | R | 566.2 | 62.25 | 2.43 |
| Pat#21 | NR | 0.5 | 74.16 | 2.06 |
| Pat#22 | R | 1676.9 | 7.00 | 0.00 |
| Pat#23 | NR | 0.0 | 8.80 | 0.50 |
| Pat#24 | R | 0.0 | 18.70 | 0.49 |
| Pat#25 | NR | 0.0 | 26.40 | 1.82 |
| Pat#26 | R | 1203.8 | 73.70 | 2.17 |
| Pat#27 | R | 168.8 | 76.80 | 2.28 |
| Pat#28 | NR | 0.0 | 71.70 | 2.23 |
| Pat#29 | NR | 3.7 | 83.90 | 2.07 |
| Pat#30 | NR | 6.7 | 38.52 | 0.24 |
| Pat#31 | NR | 1.5 | 83.90 | 1.47 |
| Pat#32 | R | 2311.7 | 83.90 | 2.04 |
| Pat#33 | NR | 96.4 | 61.25 | 1.91 |
| Pat#34 | NR | 0.0 | 38.65 | 2.28 |
| Pat#35 | NR | 0.0 | 32.83 | 0.00 |
| Pat#36 | R | 193.6 | 9.96 | 2.19 |
| Pat#37 | R | 361.1 | 60.69 | 1.47 |
| Pat#38 | R | 386 | 43.03 | 1.55 |
| Pat#39 | NR | 7.7 | 45 | 1.26 |
| Pat#40 | NR | 383.9 | 41.43 | 1.31 |
