# Supplementary material for: A specific anti-citrullinated protein antibody profile identifies a group of rheumatoid arthritis patients with a toll-like receptor 4-mediated disease
Source: Arthritis Res Ther. 2016 Oct 6;18:224. doi: 10.1186/s13075-016-1128-5 (PMC5053084; doi:10.1186/s13075-016-1128-5)
Supplement: Additional file 10: — Stimulation with TLR2 and TLR4 ligands. Interfering with TLR4 signaling alone is sufficient to block synergistic IL-6 induction from monocytes stimulated by the combined TLR2 and TLR4 activation. (DOCX 84 kb) [file 13075_2016_1128_MOESM10_ESM.docx]

**Additional file 10**

**Additional file 10:** Stimulation with TLR2 and TLR4 ligands. Monocytes were isolated as described above from healthy volunteers (n=4). Cells were stimulated with suboptimal concentration of TLR2 (Pam3CSK4, 40 ng/ml, InvivoGen) and TLR4 (LPS, 10 pg/ml) ligands, alone or in combination. Where applicable, monocytes were also preincubated (30 min, 10 μg/mL) with NI-0101 or the isotype control before stimulation. After 24 hours of stimulation, supernatants were harvested and IL-6 production assessed by ELISA. Results are presented as the mean ± SEM of the fold increase with the combination of LPS plus Pam3SCK4 over the sum of the response to the two individual ligands. Each condition was tested in triplicate. Mann Whitney’s U test was performed to compare changes observed. ** p < 0.01, *** p < 0.001.
