# Supplementary material for: A specific anti-citrullinated protein antibody profile identifies a group of rheumatoid arthritis patients with a toll-like receptor 4-mediated disease
Source: Arthritis Res Ther. 2016 Oct 6;18:224. doi: 10.1186/s13075-016-1128-5 (PMC5053084; doi:10.1186/s13075-016-1128-5)
Supplement: Additional file 11: — cFb-IC-induced TNF-α production was inhibited by TLR4 blockade with NI-0101. Fc-dependent mechanism of action rendered NI-0101 a more effective antibody in inhibiting cFb-IC-stimulated TNF-α production from macrophages. (DOCX 150 kb) [file 13075_2016_1128_MOESM11_ESM.docx]

**Additional file 11**

**Additional file 11**. cFb-IC induced TNF-α production was inhibited by TLR4 blockade with NI-0101. Blood-derived macrophages from healthy volunteers were left untreated or preincubated with 10 μg/mL NI-0101, NI-0101 D265A, human IgG_1_ isotype control or human IgG_1_ D265A isotype control for 30 min before the addition of cFb-IC. cFb-IC were prepared by using coated citrullinated human fibrinogen and rabbit anti-fibrinogen polyclonal antibody. Each condition was tested in triplicate. Supernatants collected after 24 h were analyzed for TNF-α by ELISA. Results in the figure are representative of at least two independent experiments. Data are represented as mean ± SEM. Mann-Whitney’s U test was performed to compare the different groups: *** p < 0.001, ** p<0.01.
